# Drug-Induced IgA Vasculitis in an Adult

**DOI:** 10.7759/cureus.34270

**Published:** 2023-01-27

**Authors:** Miranda Yousif, Neil H Vigil, Reem Haddad

**Affiliations:** 1 College of Medicine, University of Arizona College of Medicine - Phoenix, Phoenix, USA; 2 Dermatology, HonorHealth, Scottsdale, USA; 3 Department of Dermatology, Carl T. Hayden Veterans’ Administration Medical Center, Phoenix, USA

**Keywords:** iga vasculitis, small-vessel vasculitis, drug-related adverse event, adverse drug event, use of antibiotic, cutaneous vasculitis

## Abstract

A 65-year-old man developed palpable purpuric papules and plaques on his lower extremities, which quickly spread to his trunk and upper extremity after being prescribed cephalexin and doxycycline in the emergency room. Here, we define the details of a textbook-like presentation of IgA vasculitis, formerly referred to as Henoch-Schönlein purpura, in an adult.

## Introduction

Immunoglobulin A vasculitis (IgAV), formerly referred to as Henoch-Schönlein purpura, is a systemic vasculitis caused by immune-complex deposition in small vessels leading to leukocytoclastic vasculitis that clinically presents with palpable purpura and abdominal and joint pain.

IgAV is most common in children with an annual incidence of 10-20 cases per 100,000 [1]. Although an estimated 90% of cases occur at 2-10 years of age, adults can also develop this condition, rarely after exposure to antibiotics [1]. Current literature suggests that for every 150-200 reported cases in children, there is one reported case in adults. Moreover, while the prevalence in the adult population is unknown, the incidence is estimated to be one in 1,000,000 [2].

The prognosis for both children and adults is overall favorable, with spontaneous resolution reported to be 94% and 89%, respectively [3]. Thus, treatment is generally supportive care with monitoring for further complications. Diagnostic criteria for IgAV include clinical suspicion of palpable purpura, arthralgias, and potential organ involvement (specifically, abdominal pain, hematuria, and proteinuria), along with histological evidence of leukocytoclastic vasculitis and IgA immune-complex deposition in glomerular or vessel walls [4]. After a confirmed diagnosis, further testing is necessary to assess organ involvement, primarily renal and gastrointestinal systems.

Currently, treatment guidelines are not well established for IgAV. Successful treatments with immunosuppressants and high-dose steroids in severe disease have been reported in case reports [2] and rapid evidence reviews [3]. Short and long-term prognoses depend on the severity of gastrointestinal and renal involvement, respectively [3]. For patients with more severe diseases, close follow-up and monitoring of renal function, urinalysis, and blood pressure are helpful in identifying delayed complications.

## Case presentation

A 65-year-old male with a medical history significant for type 2 diabetes mellitus, morbid obesity, and venous insufficiency presented to the emergency room for evaluation of a chronic diabetic foot ulcer. On evaluation, he was afebrile with normal vital signs. Examination showed a tender unilateral chronic foot ulcer. His laboratory values were significant for a mild leukocytosis of 13.2 and normal C-reactive protein. He was empirically started on a treatment course of oral cephalexin and doxycycline.

**Figure 1:**
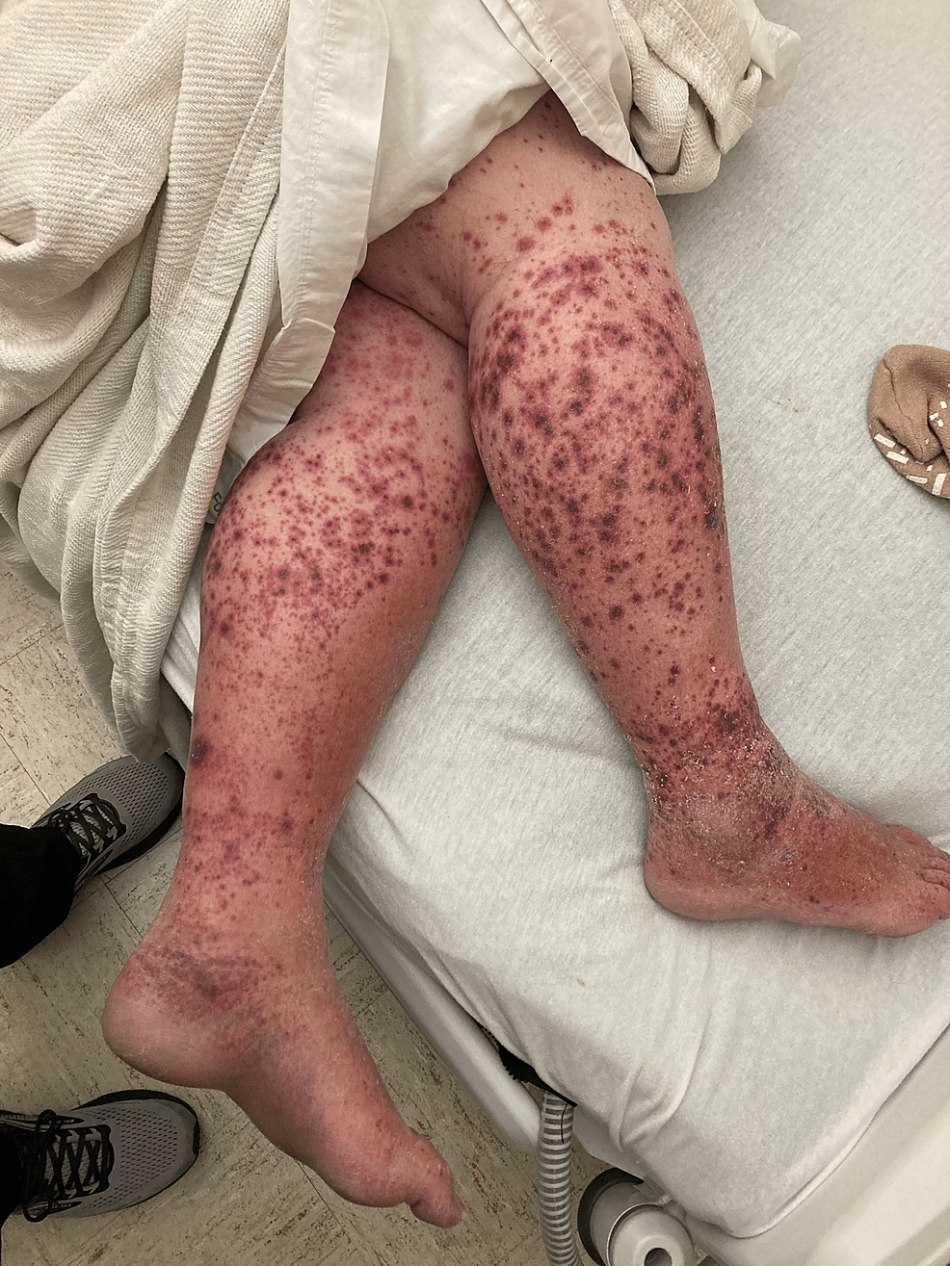
Palpable purpuric lesions appreciated on the patient’s lower extremities.

**Figure 2:**
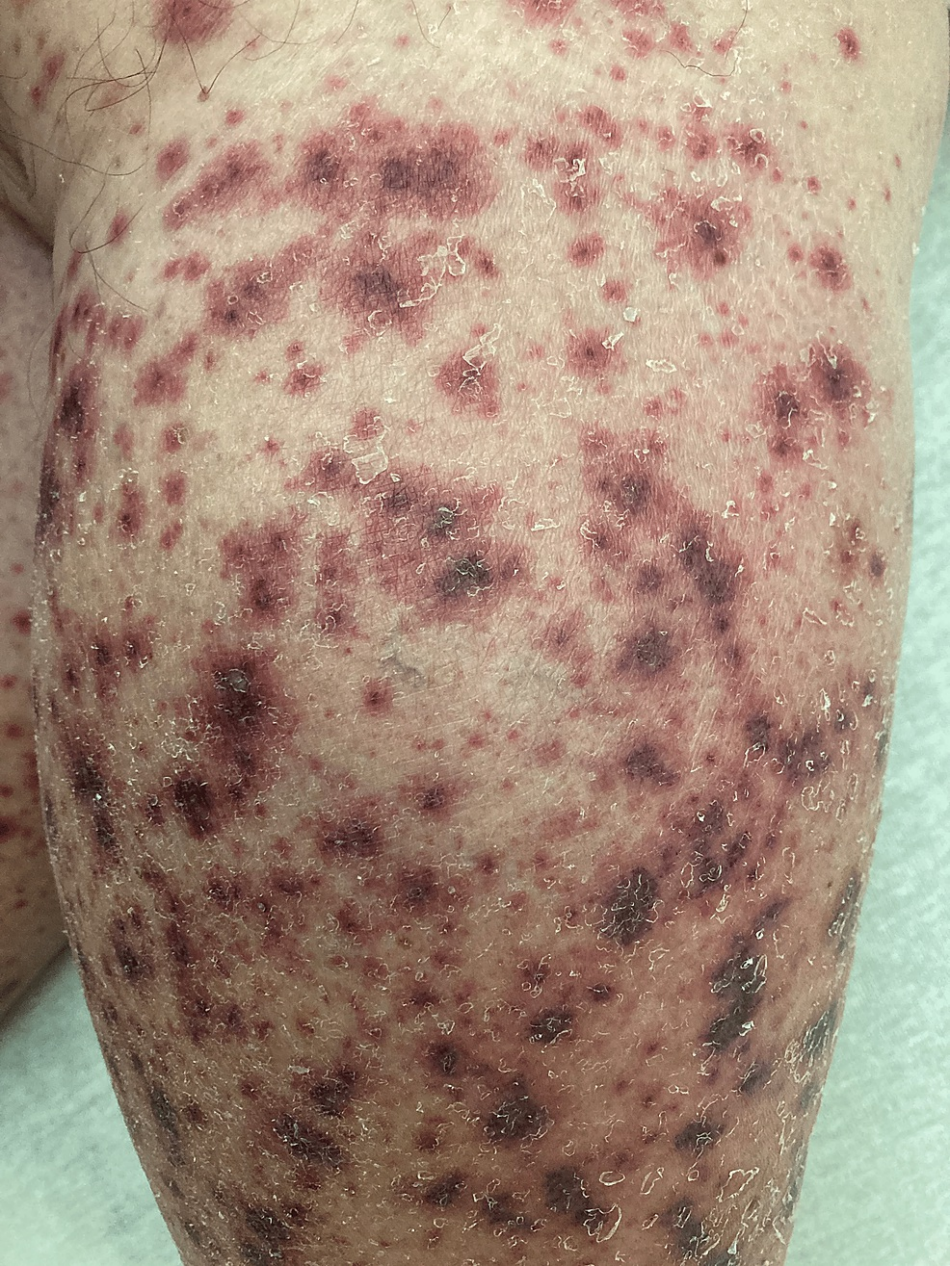
A closer image of painful and pruritic plaques.

Eight days later, the patient noted scattered pruritic and painful petechiae on his lower extremities (Figure 1). These petechiae erupted over 48 hours into palpable purpuric papules and plaques involving the legs and to a lesser degree the buttocks, abdomen, and forearms (appreciable in Figure 2), prompting the patient to return to our facility for admission. On admission (day 11 of cephalexin and doxycycline), his antibiotics were discontinued. Two punch biopsies of lesions from his left forearm were obtained and sent for hematoxylin and eosin (H&E) stain and direct immunofluorescence (DIF). Vitals, creatine levels, and white blood cell counts were unremarkable. A urinalysis revealed proteinuria of 30 mg/dL and small amounts of hematuria (five red blood Cell units). An extensive serologic workup was initiated for suspected small-to-medium-vessel vasculitis. Pertinent findings included undetectable antistreptolysin O (ASO) titer and negative antinuclear antibodies, antineutrophil cytoplasmic antibodies, and hepatitis B/hepatitis C antibodies. A nasal swab for methicillin-resistant *Staphylococcus aureus* was negative. Laboratory tests revealed normal complement levels (C3/C4/CH50).

H&E staining of his forearm lesion revealed orthokeratosis overlying the epidermis with slight spongiosis. The adjacent dermis was notable for perivascular and interstitial infiltrates of neutrophils accompanied by many extravasated red blood cells, eosinophils, and nuclear debris. Neutrophils had permeated small and medium vessels. DIF revealed granular IgA deposits and much weaker C3, C5b-9, and fibrin deposition in and around the superficial papillary dermal blood vessels confirming our suspected diagnosis of IgAV.

The constellation of clinical symptoms, physical examination findings, and histologic findings including DIF supported our diagnosis of IgAV.

Within two days of discontinuation of antibiotics, the patient’s rash and symptoms showed notable improvement. He was managed with supportive care including compression stockings and leg elevation. During his admission, a serial evaluation showed no evidence of renal involvement, and the patient was discharged in good health. Given his clinical course and significant improvement within 48 hours of discontinuing antibiotics, negative ASO titer, and lack of preceding upper respiratory infection, we posit cephalexin and doxycycline as the most likely precipitating cause of the patient’s IgAV.

## Discussion

IgAV is an uncommonly reported systemic vasculitis. Palpable purpura is the most common cutaneous manifestation that can be associated with gastrointestinal, renal, and rheumatological involvement.

Guidelines for the management of adult-onset IgAV are sparse. There is still much debate about the role and efficacy of glucocorticoids and immunosuppressants in treatment as most findings are limited to the pediatric population. However, recent reviews have shed light on emerging therapies such as cyclophosphamide, rituximab, and medications implicated in the treatment of IgA nephropathy and call for more randomized control trial data [5,6].

Some clinicians recommend early diagnosis with a contrast-enhanced CT scan of the abdomen, small intestinal endoscopy, and a renal needle biopsy for earlier diagnosis, as well as a combination of antihistamines, gastric acid suppressants, and glucocorticoids to reduce gastrointestinal pain, hyperemia, and proteinuria [7].

Pediatric IgAV is more common and generally better understood. Most cases reported in children have an excellent prognosis, and generally, the development of nephritis serves as a prognostic marker for a more severe clinical course [1]. While some studies suggest systemic steroids may decrease the occurrence of nephropathy, others report steroids do not reduce the development of nephropathy in the pediatric population. However, there may be a role in long-term management with an angiotensin-converting enzyme inhibitor or angiotensin receptor blockers, as Leung et al. [1] suggest that they have the potential to prevent further glomerular injury, notable for potential use in adult medicine. Although it is believed that compared to children, adults with IgAV have a more severe systemic disease and greater kidney injury, a small retrospective study found that adult-onset disease is associated with cutaneous manifestation earlier, and adults present with higher rates of arthritis, but actually there was no significant difference in the occurrence of nephritis between adult and pediatric populations [8]. Likely attributed to similarities in renal outcomes, these researchers also reported no significant difference in the necessity of immunosuppressive medications between adults and children [8]. These results contrast the findings from a slightly larger retrospective study comparing adult and pediatric data [9]. Results suggested that adults presented with significantly more renal involvement (p = 0.02) and received significantly more steroids (p = 0.002) and immunosuppressant therapy (p = 0.016) than children, and adults developed significantly higher rates of end-stage kidney disease (p < 0.001) [9].

There is a gap in the literature about the causes of adult-onset IgAV. There are known precipitating events that may be inciting the dysregulated immune response that is histologically seen. Upper respiratory infections are thought to cause the majority of cases, and *Streptococcus *and parainfluenza virus are the predominant pathogens [10]. Some case reports highlight culprit medications, especially antibiotics (as seen in this case), that are thought to cause adult-onset IgA deposition in small blood vessels. It is currently understood that the interaction between endothelial cells and leukocytes can lead to endothelial damage, perivascular leukocytic infiltrates, and inflammatory cytokines which precede the deposition of immune complexes [10]. Pathologic deposition of IgA complexes and activation of the complement system may be suggestive of a dysregulated immune response to antigens, which may be causing the vasculitis and granulomatous reaction that is seen histologically.

## Conclusions

As with most cases of IgAV, prompt recognition and intervention allowed for an excellent prognosis for this patient. Although IgAV is uncommon in adults, physicians should be aware of this condition as well as rare drug-induced causes to allow for the prompt removal of inciting medications. Management of IgAV includes immediate discontinuation of the offending medication(s), supportive therapy for reported symptoms, and prevention and/or monitoring for organ involvement.
